# A Single Surgeon’s Long-Term Experience With Full-Thickness Skin Grafts for Nasal Defect Reconstruction

**DOI:** 10.7759/cureus.82003

**Published:** 2025-04-10

**Authors:** Harel G Schwartzberg, Mohammed S Rais, Aran Yoo, Alexander M Germann, Charles L Dupin

**Affiliations:** 1 Plastic Surgery, Louisiana State University Health Sciences Center, New Orleans, USA; 2 School of Medicine, Louisiana State University Health Sciences Center, New Orleans, USA; 3 Plastic Surgery, The Meltzer Clinic, Scottsdale, USA

**Keywords:** basal cell carcinoma, ftsgs, full-thickness skin grafts, nasal defect, nasal reconstruction, nasal tip reconstruction, skin grafting, yin yang flaps

## Abstract

Nasal reconstruction is a constantly evolving area of plastic surgery. Many nasal defects are managed with staged local or regional flaps, which often impart significant donor site morbidity and may place psychosocial stress on patients. Full-thickness skin grafts (FTSGs) are a viable option for reconstruction. When FTSGs are used, the skin is typically taken from behind or in front of the ear, though this skin does not always match the nasal skin well. This study examines the use of FTSGs taken from the forehead, specifically from the widow’s peak area, to reconstruct nasal defects.

This is a retrospective study of nasal reconstructions performed by one surgeon from 2014 to 2019. After receiving IRB approval, patient charts were reviewed. Eligible patients had shallow nasal defects (skin and subcutaneous tissue only) with good blood supply in the wound bed. All FTSGs were taken from the widow’s peak area of the forehead. Grafts were thinned, placed into the defect, and secured with sutures and a bolster dressing for one week. Donor sites were closed directly if small or repaired with Yin Yang flaps if larger. Patients were followed until the site was fully healed, and outcomes focused on graft survival, cosmetic appearance, and complications.

Twenty patients met the criteria for the study; 60% were male with an average age of 74. Most (65%) had basal cell carcinoma, and the nasal tip was the most common site repaired (12 cases). The average defect size was 1.69 cm (range 0.5-4.5 cm). Cosmetic results were generally favorable, with good color, thickness, and texture matching.

This study shows that FTSGs from the forehead’s widow’s peak offer a reliable and cosmetically favorable option for nasal reconstruction, especially for lower third defects of the nose. Compared to traditional flaps or ear skin grafts, forehead FTSGs provide better skin thickness and contour match and avoid the need for multiple surgeries. This makes them an appealing option for patients who want a single-stage procedure with a quick recovery.

## Introduction

Nasal defects are notoriously difficult to reconstruct aesthetically due to varying skin thickness, texture, and contours of the nose [1,2]. Traditionally, most nasal defects have been reconstructed via local and regional flaps to replace 'like with like.' However, these options may involve considerable donor site morbidity, inconvenience, and discomfort for patients. Additionally, many of these methods involve multiple surgeries, increasing the overall morbidity and the opportunity for complications associated with the reconstruction. Skin grafting is a surgical option with limited indications in nasal reconstruction; however, it involves little donor site risk. Previous studies have described full-thickness skin grafts (FTSGs) from various donor sources for small defects involving the upper two-thirds of the nose [3]. The most common source of FTSGs for nasal defects has traditionally been either pre- or post-auricular skin. In our study, we used an FTSG from the widow’s peak area of the forehead. In the author’s experience, this donor site matches the color, contour, and thickness, which is more comparable to the surrounding nasal soft tissue when compared to periauricular FTSGs. This makes intuitive sense, as the most common procedure used for the reconstruction of the caudal nose is the paramedian forehead flap, which also utilizes skin from the forehead [4]. This study aims to further explore the use of FTSGs harvested from the forehead to reconstruct nasal defects, mostly those of the lower third of the nose. Indications, surgical techniques, and results are discussed in this article.

## Materials and methods

After institutional review board (IRB) approval of the study protocol, a retrospective chart review was conducted, including procedures performed from 2014 to 2019 by a single surgeon. Patients were selected for FTSG nasal reconstruction if there was sufficient soft tissue present at the base of the defect and the perichondrium had not been violated. Exclusion criteria included patients whose wounds involved extensive cartilage exposure and were not candidates for skin grafting, as these wound beds are poorly vascularized and do not provide adequate support for skin grafts.

Included patients underwent FTSG nasal reconstruction if there was sufficient soft tissue at the base of the defect and the perichondrium had not been violated. All FTSGs were harvested from the forehead near the hairline in the 'widow’s peak' area. Donor sites were all closed primarily if less than 1.5 cm or with Yin Yang flaps if larger. Recipient wound beds were prepared so that healthy, bleeding tissue was present at the wound base and edges. If wound beds appeared de-vascularized, the dermal substitute Integra was used for 2 weeks to develop healthy granulating tissue at the base of the wound. FTSGs experience 10-15% primary contraction and minimal secondary contraction [3]; therefore, skin grafts were measured to fit the defect nearly perfectly. Templates were useful to accurately measure size while considering the wound bed’s contour. All skin grafts were thinned of subcutaneous fat, then sutured to the defect using non-absorbable quilting sutures in order to prevent hematoma/seroma formation. Xeroform was then bolstered onto the defect using silk sutures going through native skin and skin graft. Bolsters were removed in one week, and grafts were dressed with bacitracin and a non-adhesive dressing.

## Results

**Figure 1:**
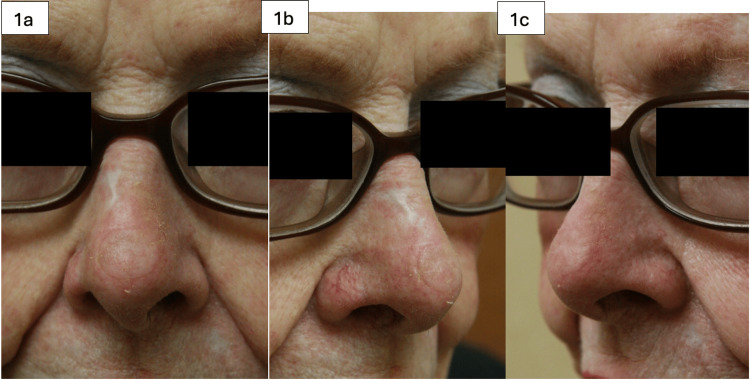
88-year-old female with a history of squamous cell carcinoma involving the nasal tip. The defect after excision measured 1.3 cm. A full-thickness skin graft (FTSG) was harvested from the forehead at the hairline. Figures 1a-1c show the reconstructed nasal tip one month post-operatively.

Twenty patients were included in this case series. Sixty percent of the patients were male, and the average age was 74.27 years. Sixty-five percent of the patients had basal cell carcinoma, while the remaining 35% had squamous cell carcinoma. Most of the patients underwent nasal tip reconstruction (N=12), while others had reconstruction of the nasal dorsum (N=2), nasal sidewall (N=1), and alar regions (N=2). The average size of the lesion, with margins excised, was 1.69 cm (range 0.5-4.5 cm). Surgical sites after the lesions were excised involved skin and subcutaneous tissue only (Table 1). Patients were followed post-operatively until their surgical site was fully healed. Occasionally, skin grafts initially appeared pale and/or developed eschars over the FTSGs (Figure 1a); however, these spontaneously separated over several weeks, and each graft had a reliably good take (Figure 1b, 1c) [3].

**Table 1:** Summary of data collected from patients undergoing full-thickness skin grafts (FTSG) for nasal defects.

Variable	Result
Number of Patients	20
Gender	60% Male
Average Age	74.3 years
Diagnosis	65% Basal Cell Carcinoma
Reconstruction Site	Nasal Tip (N=12)
Average Lesion Size (with margins)	1.69 cm (Range 0.5 - 4.5 cm)
Common Graft Appearance (Early)	Pale or Eschar Formation
Final Graft Take	95% (after eschars separated)
Cosmetic Outcome	Good color, texture, and thickness match
Complications	One case of graft failure due to hematoma (re-grafted successfully)
Overall Complication Rate	5% (1/20 patients)
Additional Procedures Performed	5% (1/20) (re-grafting)

**Figure 2:**
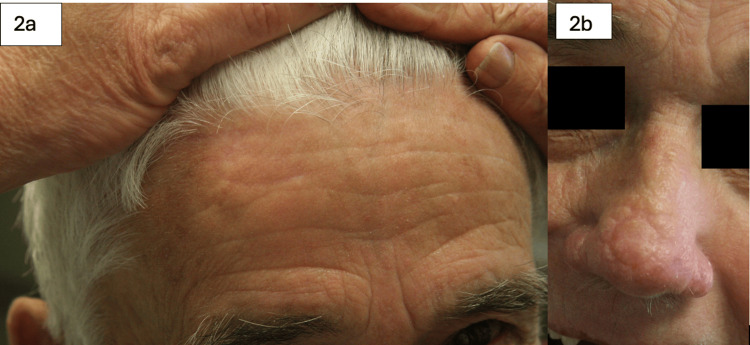
79-year-old male with a history of melanoma in-situ involving the nasal tip. The defect after excision measured 1.5 cm. A full-thickness skin graft (FTSG) was harvested from the forehead at the hairline. Figure 2a shows the reconstruction site six weeks post-operatively; Figure 2b shows the donor site well-healed at six weeks post-operatively.

**Figure 3:**
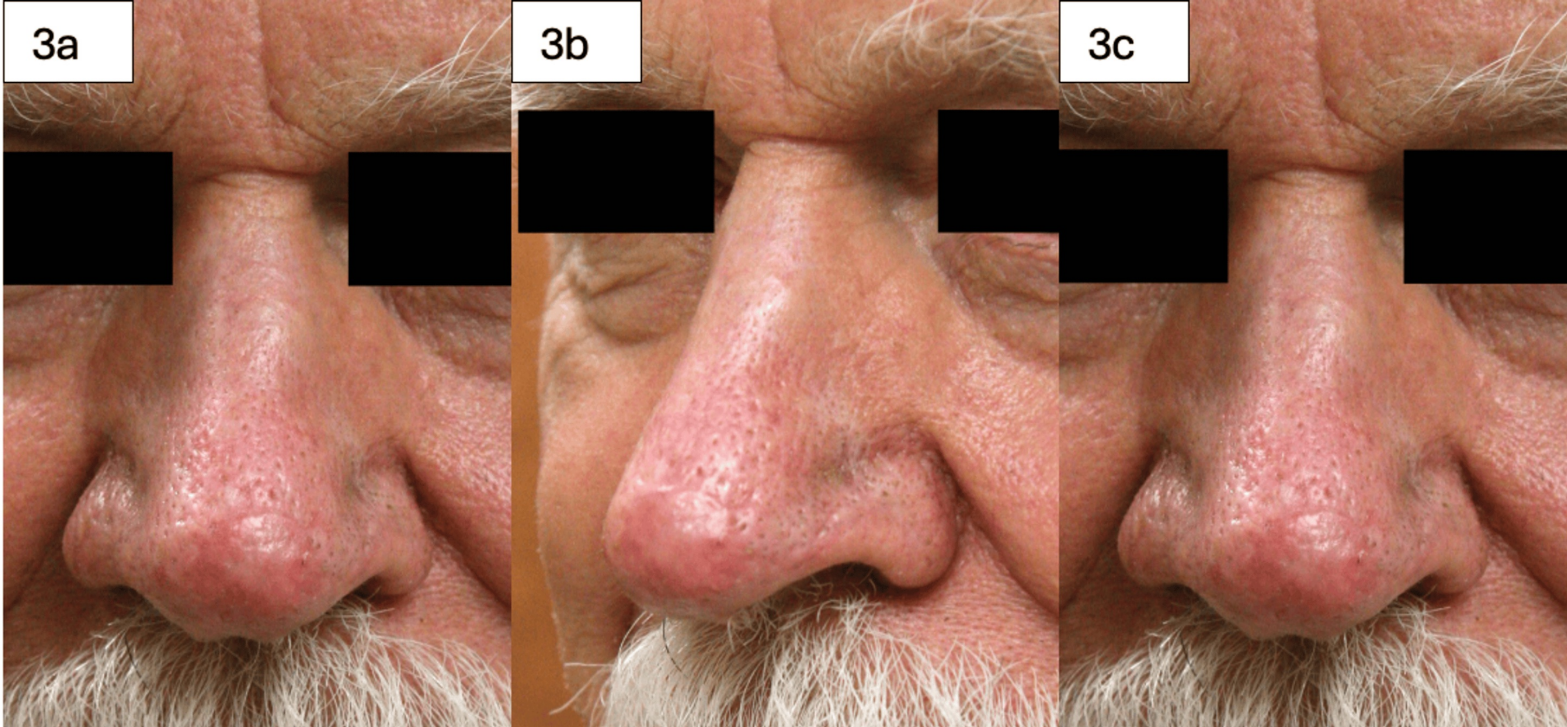
83-year-old male with a history of basal cell carcinoma involving the nasal tip. The defect after excision measured 2.7 cm. A full-thickness skin graft (FTSG) was harvested from the forehead at the hairline. Figures 3a and 3b show the graft three months post-operatively, having healed well with 100% take.

**Figure 4:**
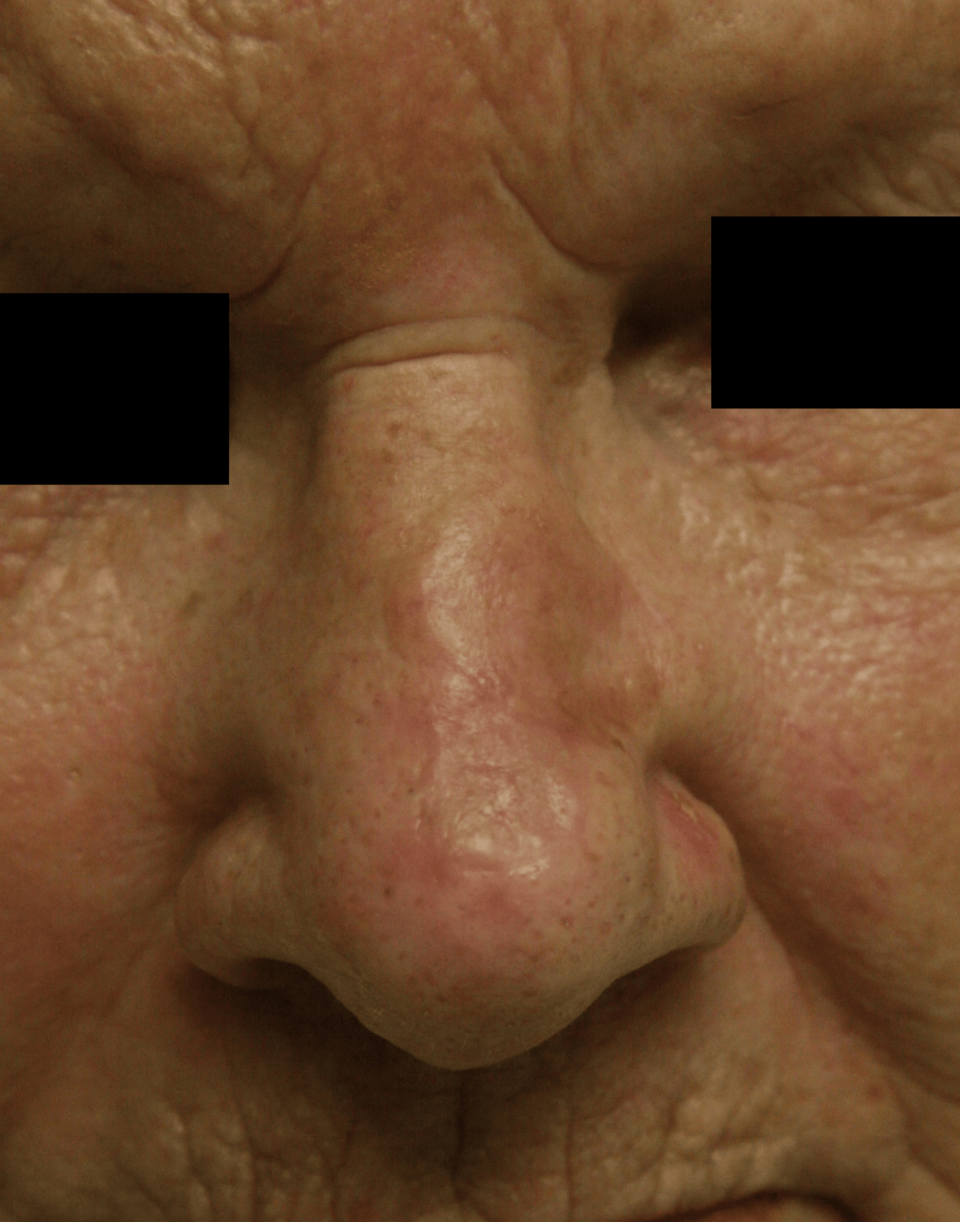
88-year-old male with a history of basal cell carcinoma involving the nasal tip and dorsum. The defect at the tip after excision measured 2.2 cm. A full-thickness skin graft (FTSG) was harvested from the forehead at the hairline. The reconstructed nasal tip, shown six months post-operatively, healed well with complete take.

**Figure 5:**
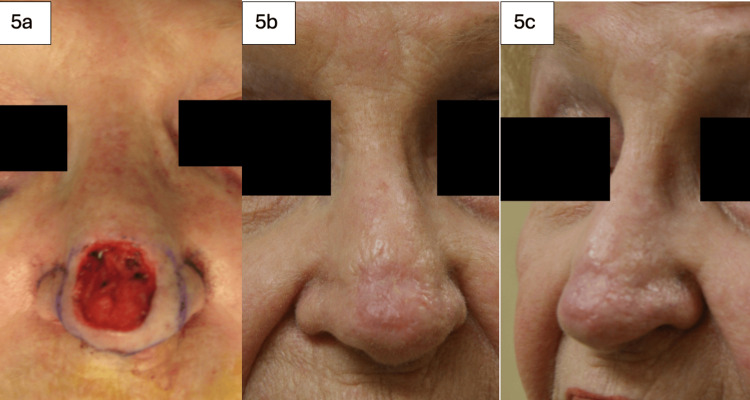
68-year-old female with a history of basal cell carcinoma involving the nasal tip. The defect after excision measured 3 cm. A full-thickness skin graft (FTSG) was harvested from the forehead at the hairline. The lesion before excision is shown in Figure 5a and immediately after excision in Figures 5b-5c. The reconstructed nasal tip, shown one month post-operatively, healed well with complete take, and the patient experienced no complications.

Most patients achieved good results in terms of cosmetic appearance and functionality. The color, texture, and skin thickness match were excellent, as demonstrated in Figures 1-5. We had one post-operative complication of graft failure, which was attributed to hematoma formation. The area was re-grafted with an FTSG taken from the forehead hairline. After the second procedure, there was a 100% graft take. There were no other complications in the patients included in this study.

## Discussion

The depth of the nasal defect is an important variable that dictates whether FTSGs are indicated. Patients with superficial soft tissue defects involving only skin and subcutaneous fat are candidates for skin grafting. Skin grafts depend entirely on the wound bed for their blood supply, and superficial soft tissue wounds tend to be well-perfused. In contrast, patients with wounds involving extensive cartilage exposure are not candidates for skin grafting, as these wound beds are poorly vascularized and will not provide adequate support for skin grafts.

The size of the nasal defect must also be considered. Small defects are defined as smaller than or equal to 1.5 cm; medium defects are 1.5 cm to 2.5 cm; large defects are greater than 2.5 cm [2,4]. Small defects are well-suited for FTSG, as loco-regional flaps provide too much bulkiness and possible pincushioning [5]. For larger defects greater than 1 cm, some authors argue that loco-regional flaps are necessary [3,5]. In this study, defects up to 4.5 cm were successfully reconstructed by skin grafting with good results. Additionally, the location of the nasal defect is a key variable. Nasal skin on the upper two-thirds of the nose is thin and mobile, while the skin on the lower third is thick, non-mobile, and composed of sebaceous glands. Traditionally, the use of FTSG was limited to the upper two-thirds of the nose, especially for nasal dorsum and sidewall. The lower third of the nose, however, is resistant to contraction and scarring, thereby providing robust underlying structural support. In this study, we demonstrated excellent results using excisions limited to the lesion and appropriate margins and FTSGs from the forehead primarily for lower third nasal reconstruction procedures. These results demonstrate a recognized principle: defect size or subunits are not strict rules for nasal reconstruction; rather, like must be replaced with like. Key goals of repairing nasal defects are to restore nasal contour, texture, and color [3].

Although previous authors have reported good results using other donor sites [6,7], we believe the forehead is an excellent donor site due to its similarity in skin texture, color, and thickness match. Given that the nose is a cosmetically sensitive area, the match of skin texture, color, and thickness are of utmost importance. The forehead FTSG fulfills all these requirements. Forehead skin has a thick compact fibro-fatty subcutaneous layer that is suitable for 'blending' into the nose tip, avoiding a depressed and patchy poor appearance [3]. Patients with deeper defects requiring reconstruction can utilize 'skin-fat grafts.' Interestingly, Kreutzer C et al. reported applying skin grafts without defatting to defects up to 83 mm with favorable results [2,8].

One study compared post-operative results between supraclavicular FTSG and local flaps (bilobed or nasolabial flaps) [9]. The study reported a 19.5% overall complication rate for 41 patients. There was no statistically significant difference in the complication rate between the two surgical methods. FTSG procedures were associated with more infections (5.4% vs. 2.6%) and partial/complete loss (8.5% vs. 5.2%). Local flaps were associated with more complications of dehiscence (5.9% vs. 2.2%) and more aesthetic deficits (6.8% vs. 2.1%). Although loss rates in this study were higher, FTSG remains an attractive reconstructive option due to little donor site morbidity. Another study comparing pre-auricular FTSG and local flaps reported that local flaps required more adjunctive procedures (triamcinolone acetonide injections and dermabrasion sessions) for aesthetic refinement. However, there was no statistically significant difference in the visual analog scale between the two groups [10].

Overall, there are several benefits to using forehead-derived FTSG for nasal reconstruction [11]. First, donor site scars are well-hidden in forehead rhytids or the hairline. Second, the forehead donor site does not restrict the use of any other reconstructive options for the patient in the future. Third, reconstruction occurs in a single procedure, allowing patients to quickly return to work and normal daily life [11,12]. In contrast, local flaps often require secondary procedures, such as flap division and thinning [8]. No secondary procedures were required for our patients, including dermabrasion for scar modification. Lastly, FTSGs are flexible enough to accommodate the subtle contours of the nose, especially of the lower third, and are less bulky compared to loco-regional flaps [13].

## Conclusions

In our case series of 20 patients, we demonstrate that FTSGs from the forehead are a viable alternative to local and regional flaps for nasal reconstruction. FTSGs restore adequate nasal contour, texture, and color while also minimizing donor site morbidity. The forehead donor site provides improved outcomes in terms of color, contour, and thickness match compared to traditionally used periauricular FTSGs. Furthermore, FTSGs allow patients to quickly return to work and resume normal daily activities. This study shows that forehead FTSGs are an excellent single-stage option for nasal reconstruction in cases that spare underlying cartilage.
